# Impact of Dual Antiplatelet Therapy with Proton Pump Inhibitors on the Outcome of Patients with Acute Coronary Syndrome Undergoing Drug-Eluting Stent Implantation

**DOI:** 10.5402/2012/692761

**Published:** 2012-06-27

**Authors:** Francesca Macaione, Carla Montaina, Salvatore Evola, Giuseppina Novo, Salvatore Novo

**Affiliations:** Division of Cardiology and Post-Graduate School of Cardiology, Center for the Early Diagnosis of Preclinical and Multifocal Atherosclerosis and for the Secondary Prevention, University Hospital “P. Giaccone” of the University of Palermo, 127-90127 Palermo, Italy

## Abstract

This study aimed to assess if proton pump inhibitors (PPIs) may reduce the effectiveness of clopidogrel, than H2 antagonist (anti-H2) in order to determine rehospitalization for acute coronary syndrome (re-ACS), target vessel revascularization (TVR) and cardiac death. This case-control study included 176 patients with ACS undergoing angioplasty (PCI) with drug-eluting stent implantation. The population was divided into two groups: PPI group (*n* = 121) consisting of patients receiving at discharge dual antiplatelet therapy (DAT) plus PPI and anti-H2 group (*n* = 55), consisting of patients receiving at discharge DAT + H2 receptor antagonist (H2RA). In a followup of 36 months the prevalence of ACS event (*P* = 0.014), TVR (*P* = 0.031) was higher in the PPI group than in the anti-H2 group; instead there was no statistically significant difference between groups for death. The variables independently associated with ACS were the diabetes, omeprazole, and esomeprazole; instead the variables independently associated with TVR were only omeprazole. Our data shows that the use of omeprazole and esomeprazole, with clopidogrel, is associated with increased risk of adverse outcomes after PCI with drug-eluting stent implantation.

## 1. Introduction

The introduction of coronary stents into clinical practice has revolutionized the treatment of coronary artery disease. The use of dual antiplatelet therapy (DAT) with aspirin and clopidogrel, in the setting of percutaneous coronary intervention (PCI) with stent implantation, is the cornerstone of the pharmacological management to prevent adverse cardiovascular events. 

In the era of drug-eluting stent prolonged antiplatelet therapy is mandatory because of the potential increased risk of late stent thrombosis [1]. Current guidelines recommend DAT for minimum of 12 months after DES implantation [2].

The obvious concern and serious complication with prolonged DAT is a bleeding of the gastrointestinal tract.

For this reason, a dual antiplatelet therapy, Clopidogrel + ASA, is commonly used with an antisecretory agent, such as proton pump inhibitors (PPIs) or H2 antagonist receptor (H2RA) [3, 4].

Intense debate is ongoing about if PPIs may reduce the efficacy and safety of clopidogrel; in fact many aspects in the *current clinical practice* are still under investigation.

Observational studies have showed inconsistency regarding whether concomitant clopidogrel and PPI use is [5–7], or not associated with adverse clinical outcomes [8–10]. 

Clopidogrel is a prodrug, belonging to the class of thienopyridines, administered orally.

About 85% of the prodrug is hydrolyzed by esterases in the blood, in an inactive carboxylic acid derivative, and only 15% of the prodrug is metabolized in the liver by cytochrome P450 with a mechanism of oxidation to generate an active metabolite. The active metabolite irreversibly inhibits platelet P2Y12 receptor for adenosine diphosphate (ADP) [11, 12]. The isoenzyme CYP2C19 plays an important role in the clopidogrel activation. Patients with reduced-function genetic polymorphisms have lower levels of the active metabolite of clopidogrel and they have an increased risk of cardiovascular events, as they have a reduced inhibition of ADP-induced platelet aggregation [13–15].

Several proton pump inhibitors (PPIs) are metabolized by CYP2C19 and thus may interact with clopidogrel metabolism [16]; therefore the use of concomitant PPIs could impede or prevent the metabolism of clopidogrel to its active metabolites through competition for the same substrate, resulting in decreased activation of clopidogrel which leads to an increased risk of adverse cardiovascular events [5, 17, 18].

The importance of this interaction arises from the large number of PCI performed annually, the increasing use of drug-eluting stent associated with long-term treatment, and the possibility of preventing an adverse interaction by avoiding coadministration of PPI.

 The rationale of this study arose from the need to further investigation about the potential interaction between proton pump inhibitors and clopidogrel.

The aim of this study was to investigate the clinical impact of PPI on the outcome of patients with acute coronary syndrome who underwent percutaneous coronary intervention with drug-eluting stent implantation, evaluating, also, the differences across the various types of IPP on the outcome.

## 2. Methods

In our retrospective study we assessed a population of 234 consecutive patients with acute coronary syndrome (ACS), as documented by electrocardiographic criteria, levels of troponin, and other clinical evidence, undergoing percutaneous coronary intervention (PCI) with drug-eluting in our Division of Cardiology, General Hospital of Palermo “Paolo Giaccone.”

Interventional procedures were performed according to international guidelines. For all patients PCI of occluded or stenotic coronary was performed by the femoral approach and use of guiding catheters 6Fr.

All patients were pretreated with aspirin and clopidogrel. The use of glycoprotein IIb/IIIa during coronary intervention was based on the operators discretion and current guidelines.

Exclusion criteria were nonaccessibility to followup, a life expectancy of less than a year, allergy to thienopyridines, ticlopidine therapy with dual antiplatelet therapy, no gastroprotective therapy, tumors, oral anticoagulation therapy, balloon angioplasty without stenting.

In the study were included the patients remaining. 

In this manner, a final sample size of 176 (75.21%) of 234 patients was enrolled. The demographic, clinical, therapeutic, and angiographic characteristics are summarized in Table 1.

All patients included had a successful procedure, defined by the recovery of coronary flow TIMI 3 (Thrombolysis in Myocardial Infarction) with residual stenosis less than 50%. After procedure, all patients were prescribed dual antiplatelet therapy, aspirin (100 mg daily) indefinitely, plus clopidogrel (75 mg/day), to continue from 12 to 18 months, with a concomitant gastroprotective therapy, using PPI or H2RA.

All patients enrolled underwent an accurate anamnesis, clinical examination, laboratory analysis, and anthropometric measurements. The database contains data listed in Table 1.

Familiar history of coronary artery disease (CAD) was defined as coronary event occurring before 55 and 65 years, for first-degree male and female, respectively.

Hypertension was defined as a blood pressure >140/90 mmHg or as use antihypertensive drugs.

Diabetes mellitus was defined as a fasting glucose ≥126 mg/dL or as use hypoglycemic drugs [19].

Patients smokersif they were current smokers or had stopped smoking since less than a year.

Dyslipidemia was defined as plasma triglycerides >150 mg/dL and/or plasma LDL-C >130 mg/dL or plasma HDL-C <40 mg/dL in men and <50 mg/dL in women. Obesity was defined as a BMI (body mass index) ≥30 kg/m^2^. Anemia was defined as hemoglobin level <12 g/dL in women and <13 g/dL in men [20].

Chronic renal failure was defined as an estimated glomerular filtration rate (eGFR) <60 mL/minute/1.73 m assessed by the MDRD equation [21].

Multivessel disease was defined as two or more lesions >50% in two or more epicardial coronary arteries. The clinical endpoints were new rehospitalization for acute coronary syndrome (re-ACS), target vessel revascularization (TVR), and cardiac death. TVR was defined as any percutaneous or surgical revascularization of any segment of the target vessel.

The target vessel is defined as the entire major coronary vessel proximal and distal to the target lesion, which includes upstream and downstream branches and the target lesion itself.

The diagnosis of ACS was based on the presence of at least two of these criteria: clinical symptoms suggestive of ACS [22], ischemic electrocardiographic changes (transitory or persistent ST segment deviation of the least 0.1 mV in at least two contiguous leads) [23], positive biomarkers of myocardial necrosis [24] (cardiac troponin, creatine kinase MB) in two consecutive determinations.

Followup of clinical end points was conducted for up to 36 months after PCI.

The clinical follow-up data were collected through telephone calls, outpatients cardiologic visit or further rehospitalization after the index procedure, and hospital records.

During follow-up periods, the patients who died for noncardiac causes were censored.

**Statistical Analysis**

Continuous variables are presented as mean ± standard deviation and were compared using Student's unpaired test. Categorical variables are presented as counts and percentages and were compared with the “Chi-square” test when appropriate (expected frequency >5). Otherwise, the Fisher's exact test was used.

Multivariate analyses (logistic regression) were conducted to identify the possible variables independently correlated with ACS and TVR in the followup. Models were developed with stepwise techniques, and by consideration all variables at univariate analysis showed *P* value ≤0.10. Results of this model were presented as odds ratio (OR) and 95% confidence intervals (95% CI) for OR.

*P* value equal or less than 0.05 was considered statistically significant.

The statistical analysis was performed using MedCalc software version 11.3.0.0.

## 3. Results

The study population consisted of 176 patient with mean age of 64.31 ± 10.08 years most of the patients were male (82.38%).

The patient sample was divided up into two groups: patients treated with proton pump inhibitors, PPI group, and patients treated with H2RA, anti-H2 group.

The PPI group included a total of 121 (68.75%) patients with mean age 63.66 ± 10.56 years.

The anti-H2 group included a total of 55 (31.25%) patients with mean age 65.75 ± 8.85 years.

**Figure 1:**
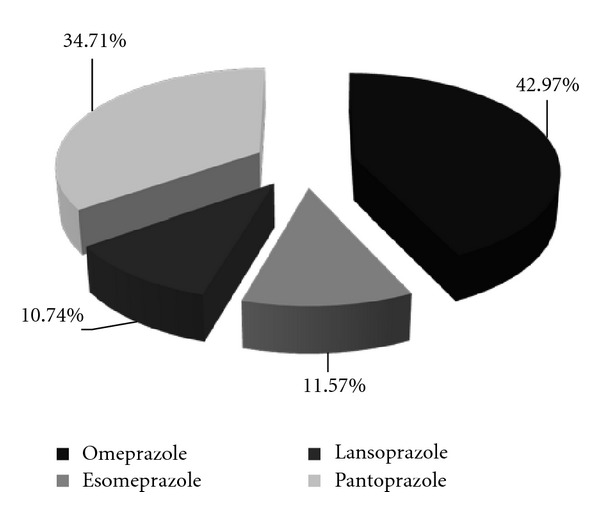
Prevalence of proton pump inhibitors in the PPIs group.

In the PPI group we included esomeprazole (*n* = 14, 11.57%), omeprazole (*n* = 52, 42.97%), lansoprazole (*n* = 13, 10.74%), and pantoprazole (*n* = 42, 34.71%) because they were the most prescribed as gastric protection in our Unit. The distribution of PPI use is shown in Figure 1.

The demographic, clinical, therapeutic, and angiographic characteristics of the population and of the groups are summarized in Table 1.

The patients of PPI group had significantly higher proportion of anemia (*P* = 0.019) and previous CABG (*P* = 0.048) compared with patients of anti-H2 group. 

There were no significant differences for all other variables and two groups did not differ with regard to medications use.

At the end of three-year followup among the patients of PPI group, there were 38 (31.40%) re-ACSs, 25 (20.66%) TVRs, 2 (1.65%) cardiac deaths.

Instead among the patients of anti-H2 group, there were 7 (17.72%) re-ACSs, 3 (5.45%) TVRs, 0 cardiac deaths.

On data obtained from followup we applied a univariate analysis that showed a higher incidence of re-ACS events (*P* = 0.014) and TVR (*P* = 0.031) in the PPI group in comparison with anti-H2 group.

An overview of events between two groups is shown in Table 2.

We also used univariate analysis to search for possible significant differences in baseline demographic, clinical, therapeutic, and angiographic parameters between the groups of patients with or without re-ACS (Table 3), with or without TVR (Table 4) and the clinical events registered in followup.

Particularly we evaluated the PPI individually such as omeprazole, esomeprazole, lansoprazole, and pantoprazole.

We found that patients with ACS presented a significant prevalence of obesity (*P* = 0.03), diabetes (*P* = 0.022) and significant prevalence of treatment with omeprazole (*P* = 0.002), esomeprazole (*P* = 0.012), and pantoprazole (*P* = 0.003); in contrast, the patients without re-ACS were a significantly higher proportion of treatment with ranitidine (*P* = 0.014).

The patients with TVR presented a significant prevalence of anemia (*P* = 0.015) and significant prevalence of treatment with omeprazole (*P* = 0.018); in contrast, the patients without TVR were a significantly higher proportion of treatment with ranitidine (*P* = 0.019).

**Figure 2:**
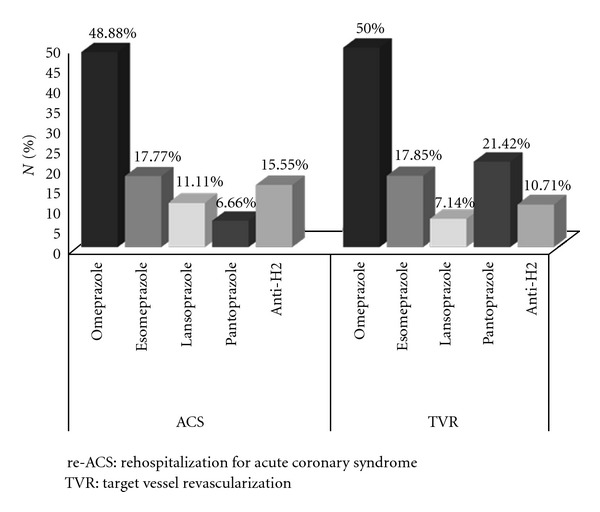
ACS and TVR events correlated at the use of PPI individually and of anti-H2. re-ACS: rehospitalization for acute coronary syndrome; TVR: target vessel revascularization.

An overview of re-ACS and TVR events correlated at the use of PPI individually and of anti-H2 is shown in Figure 2.

In addition we used logistic regression analysis in order to identify the independent predictor for occurrence of re-ACS and TVR during followup. 

Multivariate logistic regression analysis that was adjusted for all variables at univariate analysis showed *P* value ≤0.10, and we found that the omeprazole (*P* = 0.0001, OR: 5, 95% CI: 2.26–11.10), esomeprazole (*P* = 0.0016, OR: 7.09, 95% CI: 2.10–23.80), and diabetes (*P* = 0.024, OR: 2.37, 95% CI: 1.12–5.05) can be considered independent re-ACS predictors; instead only omeprazole (*P* = 0.012, OR: 2.89, 95% CI: 1.27–6.62) can be considered independent of TVR predictors (Table 5).

## 4. Discussion

Dual antiplatelet therapy (DAT) with aspirin and clopidogrel is the cornerstone of the pharmacological management in patients with acute coronary syndromes or those undergoing percutaneous coronary intervention (PCI) [25–27]. Although beneficial in these settings, prolonged DAT might be associated with the risk of gastrointestinal bleeding, [28]. For this reason, proton pump inhibitors (PPIs) and H2 antagonist are often prescribed in patients with DAT. 

Our study aims to better understand if the type of gastroprotective therapy could influence the outcome of patients, independently of some variables such as duration of treatment and the type of stent implanted. We analyzed only the patients who underwent DES implantation because in these patients the therapy with DAT after PCI is prolonged, and in this manner the patients are more exposed to treatment of gastric protection with DAT.

Our small, single center study shows that patients, discharged on clopidogrel and PPI after undergoing PCI with drug eluting stent implantation for ACS, were at a significantly higher risk of readmission for new cardiovascular adverse events. By multivariate analysis, we found that not the entire class of PPI must be incriminated but that among PPIs, omeprazole (*P* = 0.0001, OR: 5, 95% CI: 2.26–11.10) and esomeprazole (*P* = 0.0016, OR: 7.09, 95% CI: 2.10–23.80) were independent re-ACS predictors, while only omeprazole (*P* = 0.012, OR: 2.89, 95% CI: 1.27–6.62) was independent TVR predictor at 3-year followup. 

Previously described biological mechanisms support the findings of our study. Clopidogrel is converted to its metabolite by sequential oxidative steps in the liver by CYP450 isoenzymes, primarily CYP2C19. PPIs are metabolized by the same hepatic cytochrome, CYP2C19; therefore the use of concomitant PPIs could impede or prevent the metabolism of clopidogrel to its active metabolites through competition for the same substrate [5, 17, 18].

Intense debate is ongoing about if PPIs may reduce the efficacy and safety of clopidogrel.

Studies, such as the study of O'Donoghue et al., the recent works of Simon et al. [8, 29] and data from two other trials, PRINCIPLE-TIMI 44 and TRITON-TIMI 38, seem to contradict that the association between clopidogrel and PPI increases risk of adverse outcomes [30, 31]. Especially the recent study of Rossini et al. [32] has showed that association of clopidogrel and PPIs after drug-eluting stent implantation seems safe. 

Other studies, instead, suggest that PPIs decrease the effectiveness and safety of clopidogrel [5, 33, 34]. Ho et al. [5] found that the concomitant use of clopidogrel and PPIs was associated with a higher risk of death or rehospitalization for acute coronary syndrome. Gaglia et al. have examined the effect of PPI at discharge after PCI with DES on the incidence of major adverse events (MACEs) in patients with clopidogrel and with or without a PPI. Univariate survival analysis of the outcomes showed a greater rate of MACE (*P* = 0.008) and overall mortality (*P* = 0.02) in the PPI group. After multivariate analysis, the adjusted MACE hazard ratio for PPI at discharge was 1.8 (95% confidence interval 1.1 to 2.7, *P* = 0.01) [35]. Gilard et al., using a novel surrogate marker (vasodilator-stimulated phosphoprotein platelet reactivity index or PRI) for cardiovascular events, have reported that there is higher PRI in patients taking clopidogrel plus PPI than in those taking clopidogrel without PPI [36].

The findings of our study show that the PPIs, especially esomeprazole and omeprazole, lessen the efficacy and safety of clopidogrel; in fact in our study, lansoprazole is not responsible, both with a univariate analysis and with multivariate analysis, for the events considered; instead the therapy with pantoprazole if univariate analysis was significant for ACS, in multivariate analysis pantoprazole is not an independent predictor for ACS.

Several randomized trials, in fact, showed that inhibition of CYP2C19 changes within the class of proton pump inhibitors. Juurlink et al. [6] found that current use of PPIs other than pantoprazole, among omeprazole, lansoprazole, and rabeprazole, in elderly patients on clopidogrel was associated with a significantly increased short-term risk of reinfarction after acute myocardial infarction. Angiolillo et al. in their study have shown that a “Drug-drug” interaction exists for clopidogrel and omeprazole but not for clopidogrel and pantoprazole [37]; other studies have shown that pantoprazole is less potent than omeprazole to inhibit CYP2C19 [38] and seems not to interfere in the pharmacodynamic of clopidogrel. Sibbing et al. [7] have shown that attenuating effects of concomitant PPI treatment on platelet response to clopidogrel were restricted to the use of omeprazole. No attenuating effects on platelet response to clopidogrel were observed for pantoprazole or esomeprazole. In vitro tests showed that, among PPIs, esomeprazole and omeprazole are the most potent inhibitors of CYP2C19; on the other hand in Europe, the European Medicines Agency (EMEA) also issued a statement that concomitant use of omeprazole and other CYP2C19 inhibitors, such as esomeprazole, should be avoided in patients treated with clopidogrel [39–41].

Despite these data, the current clinical evidence does not indicate that one PPI is clearly different from another, so merely switching PPIs cannot be viewed as sufficient to avoid any potential risk.

## 5. Study Limitation 

Our study carries the inherent limitations of an observational study, including failure to account for all confounding variables that could have contributed to the observed findings. Other study limitations are the small study population, telephone followup, the possible reduced absorption of the intestinal mucosa, the presence of residual confounding in the association and especially the contribution of selection bias which may be due to the presence of subjects presenting a polymorphism of CYP2C19.

Different studies have already demonstrated that a CYP2C19 gene polymorphism is associated with a higher platelet aggregation and an increase in adverse cardiac events similar to the poor antiplatelet effects of omeprazole or esomeprazole [38] on clopidogrel. High platelet activity linked to inhibition of PPIs on clopidogrel and a no-response to clopidogrel linked to a polymorphism of cytochrome P450 are associated with an increased risk of adverse events after stent implantation [42, 43].

## 6. Conclusion

In conclusion, our findings suggest that omeprazole and esomeprazole, in combination with clopidogrel, have been associated with a lower efficacy of clopidogrel in patients with ACS undergoing coronary stenting with drug-eluting stent implantation. This has not been found with other PPIs. 

Pending further evidence, we discourage the concomitant use of omeprazole or esomeprazole, as a routine prophylactic, how many times it is used, with the clopidogrel, and we suggest to use anti-H2 or another PPI as lansoprazole or pantoprazole, such as gastric protection, with DAT assessing the possible onset of gastric disorders. However the question of whether the efficacy of clopidogrel is influenced by concomitant use of PPI is open due to the fact that the studies conducted had conflicting results.

Research now in progress will almost certainly help clarify the picture.

## Figures and Tables

**Table 1:** Population characteristics.

	Total (*n* = 176)	PPI group (*n* = 121)	Anti-H2 group (*n* = 55)	*P* value < 0.05
Demographics characteristics				
Age	64.31 ± 10.08	63.66 ± 10.56	65.75 ± 8.85	0.203
Male *n* (%)	145 (82.38)	97 (80.16)	48 (87.27)	0.196
Clinical characteristics *n* (%)				
Smoke	61 (34.65)	45 (37.19)	15 (27.27)	0.265
Dyslipidemia	97 (55.11)	65 (53.71)	32 (58.18)	0.697
Familiar history	63 (35.79)	42 (34.71)	21 (38.18)	0.782
Hypertension	130 (73.86)	85 (70.24)	45 (81.81)	0.152
Obesity (BMI > 30 Kg/m^2^)	22 (12.50)	11 (9.09)	11 (20)	0.074
Diabetes	78 (44.31)	50 (41.32)	33 (49.09)	0.43
Previous CABG	11 (6.25)	11 (9.09)	0 (0)	**0.048**
Previous AF	5 (2.84)	1 (0.82)	4 (7.27)	0.057
Previous AMI	28 (15.90)	20 (16.52)	8 (14.54)	0.912
Previous PCI	38 (21.59)	29 (23.96)	9 (16.36)	0.348
Previous HF	4 (2.27)	3 (2.47)	1 (1.81)	0.784
Renal failure	38 (21.59)	28 (23.14)	10 (18.18)	0.587
Anemia *n* (%)	62 (35.22)	50 (41.32)	12 (21.81)	**0.019**
Therapy at discharge *n* (%)				
ACE inhibitors	106 (60.22)	75 (61.98)	30 (54.54)	0.443
*β*-blockers	137 (77.84)	94 (77.68)	43 (78.18)	0.903
Statine	163 (92.61)	109 (90.08)	54 (98.18)	0.111
Angiographics data				
Multivessel disease *n* (%)	64 (36.36)	44 (36.36)	20 (36.36)	0.865
No. of stents	1.26 ± 0.56	1.24 ± 0.60	1.25 ± 0.55	0.916

CABG: coronary artery bypass graft surgery; AF: atrial fibrillation; HF: heart failure; AMI: acute myocardial infarction; PCI: percutaneous coronary intervention.

**Table 2:** Statistical analysis cardiovascular events.

	Total (*n* = 176)	PPI group (*n* = 121)	Anti-H2 group (*n* = 55)	*P* value < 0.05
re-ACS *n* (%)	45 (25.58)	38 (31.40)	7 (12.72)	0.014
TVR *n* (%)	28 (15.90)	25 (20.66)	3 (5.45)	0.031
Cardiac Death *n* (%)	2 (1.36)	2 (1.65)	0 (0)	0.84

re-ACS: rehospitalization for acute coronary syndrome; TVR: target vessel revascularization.

**Table 3:** Univariate analysis for re-ACS event.

	Patients with re-ACS (*n* = 45)	*P* value < 0.05	Patients without re-ACS (*n* = 131)
Age	66.8 ± 10.45	0.055	63.46 ± 9.85
Male *n* (%)	37 (82.22)	0.846	108 (82.24)
Smoke	16 (35.55)	0.97	45 (0.34)
Dyslipidemia	21 (46.66)	0.25	76 (58.01)
Familiar history	14 (31.11)	0.562	49 (37.40)
Hypertension	33 (73.33)	0.918	97 (74.04)
Obesity (BMI > 30 Kg/m^2^)	1 (2.22)	**0.03**	21 (16.03)
Diabetes	27 (60)	**0.022**	51 (38.93)
Renal failure	9 (20)	0.92	29 (22.13)
Anemia *n* (%)	18 (40)	0.55	44 (33.58)
Multivessel disease *n* (%)	15 (33.33)	0.756	49 (37.40)
No. Stents	1.24 ± 0.60	0.918	1.25 ± 0.55
Omeprazole	22 (48.88)	**0.0019**	30 (22.90)
Esomeprazole	8 (17.77)	**0.012**	6 (4.58)
Lansoprazole	5 (11.11)	0.43	8 (6.10)
Pantoprazole	3 (6.66)	**0.0033**	39 (29.77)
Anti-H2	7 (15.55)	**0.0144**	48 (36.64)

**Table 4:** Univariate analysis for TVR event.

	Patients with TVR (*n* = 28)	*P* value < 0.05	Patients without TVR (*n* = 148)
Age	64.92 ± 9.93	0.729	64.20 ± 10.13
Male *n* (%)	23	0.815	122
Smoke	11	0.730	50
Dyslipidemia	15	0.977	82
Familiar history	9	0.822	54
Hypertension	21	0.932	109
Obesity (BMI > 30 Kg/m^2^)	2	0.525	20
Diabetes	18	0.034	60
Renal failure	6	0.819	32
Anemia *n* (%)	16	**0.015**	46
Multivessel disease *n* (%)	12	0.57	52
No. Stents	1.26 ± 0.56	0.870	1.24 ± 0.60
Omeprazole	14	**0.018**	38
Esomeprazole	5	0.083	9
Lansoprazole	2	0.73	11
Pantoprazole	6	0.93	36
Anti-H2	3	**0.019**	52

**Table 5:** Logistic regression analysis, independent correlates of acute coronary syndrome (ACS) and of target vessel revascularization (TVR).

Variability	Coefficient	Std. error	*P* value	OR	95% CI
re-ACS					
Omeprazole	1.6113	0.4059	0.0001	5.0093	2.2606–11.1002
Esomeprazole	1.9588	0.6198	0.0016	7.09	2.1042–23.8046
Diabetes	0.8646	0.3851	0.0248	2.37	1.1159–5.0502
TVR					
Omeprazole	1.0629	0.4222	0.0118	2.8947	1.2654–6.6222

re-ACS: rehospitalization for acute coronary syndrome; TVR: target vessel revascularization.
